# Evaluation of Potential Developmental Precursors to Executive Function in Young Children with Motor Delays: Exploratory Study

**DOI:** 10.3390/bs14121201

**Published:** 2024-12-14

**Authors:** Andrea B. Cunha, Iryna Babik, Regina T. Harbourne, Stacey C. Dusing, Lin-Ya Hsu, Natalie A. Koziol, Sarah Westcott-McCoy, Sandra L. Willett, James A. Bovaird, Michele A. Lobo

**Affiliations:** 1 Munroe Meyer Institute, University of Nebraska Medical Center, Omaha, NE 68198, USA; abaraldicunha@unmc.edu (A.B.C.); swillett@coloradomesa.edu (S.L.W.); 2 Department of Psychological Science, Boise State University, Boise, ID 83725, USA; irynababik@boisestate.edu; 3 Department of Physical Therapy, Duquesne University, Pittsburgh, PA 15282, USA; harbourner@duq.edu; 4 Department of Biokinesiology and Physical Therapy, University of Southern California, Los Angeles, CA 90033, USA; stacey.dusing@pt.usc.edu; 5 Division of Physical Therapy, Department of Rehabilitation Medicine, University of Washington, Seattle, WA 98195, USA; linyahsu@uw.edu (L.-Y.H.); westcs@uw.edu (S.W.-M.); 6 Nebraska Center for Research on Children, Youth, Families and Schools, University of Nebraska-Lincoln, Lincoln, NE 68588, USA; nkoziol@unl.edu; 7 Department of Kinesiology, Colorado Mesa University, Grand Junction, CO 81501, USA; 8 Department of Educational Psychology, University of Nebraska-Lincoln, Lincoln, NE 68588, USA; jbovaird2@unl.edu; 9 Biomechanics & Movement Science Program, Department of Physical Therapy, University of Delaware, Newark, DE 19713, USA

**Keywords:** object interaction, child development, executive function, motor delays

## Abstract

This study aimed to explore whether early developmental abilities are related to future executive function (EF) in children with motor delays. Fourteen children with motor delays (*Mean age* = 10.76, *SD* = 2.55) were included from a larger study. Object interaction and developmental outcomes (Bayley-III) were evaluated at baseline and 3, 6, and 12 months post-baseline. Bayley-III and EF assessments (Minnesota Executive Function Scale) were conducted at 36 months post-baseline. Children with high EF demonstrated advanced early bimanual, visual–bimanual, receptive language, expressive language, and fine motor skills compared to children with low EF. Significant positive correlations between later Bayley-III and EF scores were found for cognitive, expressive language, and fine motor scores. These preliminary results suggest that early developmental skills support the emergence of EF in children with motor delays.

## 1. Introduction

Executive function (EF) refers to a set of skills that allow individuals to engage in goal-directed activities requiring attention, planning, conscious information processing, and problem-solving [1,2]. EF has been conceptualized as a dynamic system of interrelated skills, including attentional control (i.e., maintaining an alert state and orienting toward a specific stimulus), working memory (i.e., retaining and manipulating information over a short period of time), cognitive flexibility (i.e., switching between different goals, rules, or tasks), and inhibitory control [3,4] (i.e., suppressing a prepotent response). EF plays an important role in children’s cognitive functioning, emotional control, and social interactions [1].

### 1.1. Development of Executive Function

In typically developing children, EF emerges during the first twelve months of life: 11–12-month-old infants can successfully complete an “A not B” object permanence task [5,6]. Children’s working memory, cognitive flexibility, and inhibitory control continue to improve dramatically from 2 to 5 years [2,7]. For example, 3- to 4-year-old children can sort test cards by color and shape but tend to perseverate when the sorting rule changes [8,9], whereas children aged 5 years and older can switch rules easily [10]. Young children gradually develop the ability to ignore distractions [11], inhibit inappropriate responses [12], and eventually integrate these abilities to solve more complex cognitive tasks [13].

Previous research showed that infants with lower gestational age (<32 weeks), low birth weight (<1500 g), neonatal complications, genetic conditions, or brain injuries are at risk for EF delays [14,15]. Specifically, children born preterm exhibit deficits in EF skills such as spatial working memory (e.g., the ability to recall shapes, colors, spatial orientations, and locations) and emotional regulation during early infancy [16]. Infants with Down syndrome at 9–15 months visually engaged with objects for longer periods and had challenges with action planning compared to their typically developing peers [17]. 

At preschool and school ages, children born preterm or with low birthweight experienced difficulties with tasks involving inhibitory control, working memory, rule switching, verbal fluency, and demonstrated less strategic planning on problem-solving tasks compared to full-term peers [16,18]. While EF deficits tend to persist in children at risk for delays [18,19,20], intervention programs targeting motor skills offer a promising pathway for EF improvement. For example, structured motor interventions led to improvements in EF skills among 3–6-year-old typically developing children [21,22] and 7–9-year-olds with learning disabilities [23]. To understand the mechanisms of EF development, it is important to examine the effects of developmental factors on emerging EF skills.

### 1.2. Relations Between Developmental Factors and Executive Function

According to embodied cognition theory, children’s cognitive development is grounded in early sensorimotor experiences [24,25]. Execution of complex motor tasks (e.g., bimanual object manipulation, artifact construction, tool use) requires anticipatory action planning, maintaining goal-directed activity, inhibiting attention to task-irrelevant information, and making flexible adjustments of behavior to changing conditions [26]. Consequently, early sensorimotor experiences, such as postural control, locomotion, manual exploration, and the integration of sensory and motor systems, provide ample opportunities for goal-directed exploration of the environment and learning. These experiences form a foundation for the development of children’s problem-solving and cognitive skills important for EF development [27,28,29,30]. Indeed, enriched object interaction and sensorimotor experiences positively impact children’s cognitive development [29,30,31]. By contrast, delays and impairments in object exploration may negatively affect the EF skills of selective and sustained attention, goal-directedness, anticipatory motor planning, and understanding of cause–effect relations [32,33].

Previous research showed significant relations between motor and EF skills. Better fine motor skills were associated with more advanced EF in typically developing school-aged children [34,35]. Cognitive development in typically developing children was linked to improved visual processing and fine manual control [34], whereas inhibitory control and working memory measures were significantly related to manual dexterity scores [35]. Additionally, the ability to control objects (e.g., striking, catching, throwing, and rolling balls) was positively associated with inhibition skills, whereas locomotor skills (e.g., running, galloping, hopping, and jumping) positively related to working memory and inhibition skills in 3–6-year-old children [36]. Better motor performance (e.g., static and dynamic balance, ball skills, and manual dexterity) was linked to more advanced working memory in typically developing 5–6-year-old children [37]. More advanced developmental trajectories for motor and cognitive skills were associated with better problem-solving abilities in means–end tasks among typically developing children and those with motor delays during their first two years of life [38]. Impaired motor function was detrimental to the development of attentional control, distractibility, and inhibitory control [39]. These results suggest that children with better physical abilities may be better equipped to explore their environment and develop planning and problem-solving skills.

Language skills also showed a significant association with EF. Previous research reported correlations between language skills (e.g., receptive and expressive vocabulary and early literacy skills) and EF in preschool children [40,41], suggesting that better grasp of language may advance the development of EF [8,42]. Significant concurrent relations were reported between language and EF skills at the ages of 4, 5, and 6 years in typically developing children and children with or at risk for language deficits; however, early language skills did not predict future EF [43]. Moreover, children with language deficits have been reported to exhibit suboptimal EF [41,44]. For example, these children demonstrated poorer EF performance (e.g., working memory, fluency, inhibition, and planning) compared to their typically developing peers [45]. Poorer attention and EF were associated with language impairments in preschoolers [41]. These findings suggest that children with better communication skills may be better equipped to explore their environment and learn.

### 1.3. Current Study

Children with motor delays are often at an increased risk for delays in EF. Therefore, it is important to understand the developmental factors that may affect the development of EF skills in this population. The following research questions guided this study: (1) How do early developmental trajectories of object interaction skills (measured during the first two years of life) relate to future EF (measured at approximately 4 years of age) in children with motor delays? (2) How do early trajectories of cognitive, language, and motor skills (measured during the first two years of life) relate to future EF (measured at approximately 4 years of age) in these children? (3) What are the concurrent relations of cognitive, language, and motor skills to EF at approximately 4 years of age?

We hypothesized that early development of object interaction abilities and global skills (i.e., cognitive, language, and motor) would be positively related to later EF. We also hypothesized that children’s cognitive, language, and motor skills would be concurrently related to EF at the age of 4 years. This exploratory study aimed to investigate whether developmental abilities in the first two years of life are significantly related to future EF skills in a sample of children with motor delays. Identifying developmental precursors of EF in this population could enhance early diagnosis of EF delays and support the design of early interventions targeting foundational skills necessary for EF development.

## 2. Materials and Methods

### 2.1. Participants

This exploratory study used a convenience subsample from a larger longitudinal randomized controlled clinical trial for children with motor delays (ClinicalTrials.gov; identifier: NCT02593825). To be included in this study, a child had to be 7–16 months old, exhibit a motor delay, demonstrate spontaneous arm movements, and be able to sit with arm support for at least 3 s without being able to transition in or out of the sitting position [46]. Exclusion criteria consisted of any genetic or progressive neurologic disorders. 

The original sample included 112 infants with motor delays who were assessed at baseline and throughout the 12-month post-baseline period. Upon receiving supplemental funding, 55 children from the original sample (those still living in the area and having the same contact information) were invited for a 36-month post-baseline follow-up, conducted between 2019 and 2022. However, some children did not complete the supplemental assessment for various reasons: (1) 22 children were unable to participate due to the COVID-19 pandemic; (2) 19 children had technical issues (*n* = 3), lack of cooperation (*n* = 5), or physical or cognitive impairments that prevented them from performing in the assessment (*n* = 11). 

As a result, the participants were 14 children (8 males), 7 to 16 months prematurity-corrected age at baseline (*M* = 10.98, *SD* = 2.51), all with motor delays (i.e., children who scored >1 SD below the mean on the gross motor subscale of the Bayley Scales of Infant and Toddler Development, Third Edition (Bayley-III) at baseline) [47]. Participant testing and data processing followed the regulations set by the internal review boards overseeing the five study sites. Demographic and health-related information for participants is presented in Table 1.

### 2.2. Procedures and Measures

Fourteen participants were tested longitudinally (i.e., baseline and 3-, 6-, 12-, and 36-months post-baseline) either in their homes or in a research lab by experienced developmental researchers. All participants had their development (i.e., object interaction and global development) and executive function evaluated; note that each assessment was conducted at the age(s) for which it has been shown to be psychometrically appropriate (see below and in Table 2). Testing was performed only when children were in a positive or neutral mood (with negative vocalizations, facial expressions, and fidgety body movements identifying negative affect); when necessary, a visit was rescheduled. All testing sessions were video recorded with one frontal-lateral view for coding and scoring purposes.

#### 2.2.1. Object Interaction Development

An object interaction assessment [27,48] was conducted at baseline and at 3-, 6-, and 12-months post-baseline. Children were tested while seated in a booster seat or on the caregiver’s lap. An easily graspable toy (approximately 6 × 2”) was presented within the child’s reach for 20 s across five trials at different locations: (1) midline at hip level; (2) midline at chest level; (3) midline at eye level; (4) right side at chest level; and (5) left side at chest level. Data were aggregated across locations to assess object interaction skills across space. Videos of the assessment were coded using Datavyu software v.1.3.8 [49] to quantify the variables listed in Table 3. For each of the object interaction variables, data were normalized to the frequency of occurrences per minute by dividing the total frequency of behavior by the total assessment time in minutes [48].

#### 2.2.2. Global Development—Cognitive, Language, and Motor

Additionally, participants’ global developmental outcomes were assessed at baseline and 3-, 6-, 12-, and 36-months post-baseline using the Bayley-III cognitive (*Bayley-CG*), receptive language (*Bayley-RL*), expressive language (*Bayley-EL*), fine motor (*Bayley-FM*), and gross motor (*Bayley-GM*) subscales. Scaled Bayley-III scores [47] were used in statistical analyses to account for variability in participants’ ages at each visit.

#### 2.2.3. Executive Function 

**Figure 1:**
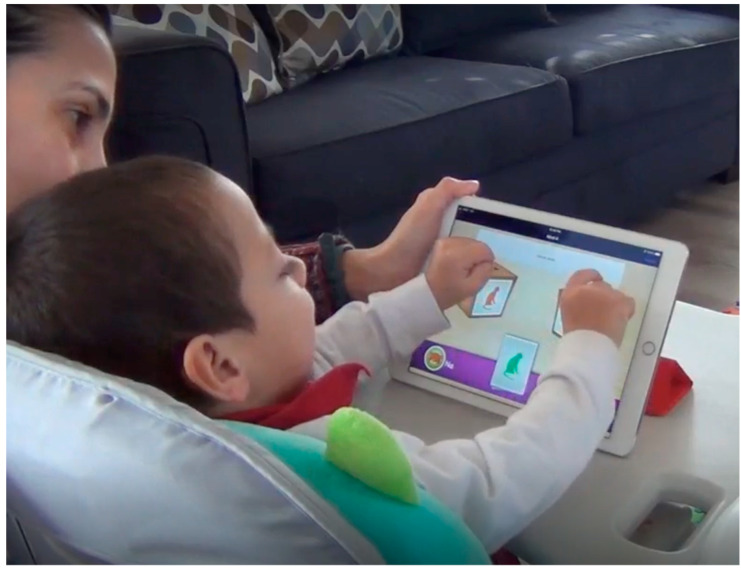
Experimental setup for the Minnesota Executive Function Scale (MEFS) at 36 months post-baseline.

Participants’ EF was measured with the Minnesota Executive Function Scale—MEFS App™ [50] at 36 months post-baseline (*M* = 48.65 months, *SD* = 3.02) using an iPad (Figure 1). The MEFS App™ is a valid and reliable game-based standardized assessment of EF skills for children aged two years and older [50,51]. The MEFS App™ assessment was administered by a trained examiner. Children were instructed to sort visual cards into one of two boxes on the screen, following an increasingly complex set of rules. Scaled *MEFS* scores were used in the statistical analyses. Additionally, for some analyses, MEFS scores were categorized (*MEFS-C* variable) into low vs. high EF (0 = low; 1 = high), using the median MEFS score (*Median* = 91) as the cutoff point. Establishing two groups based on low vs. high EF allowed us to relate longitudinal developmental trajectories of early object interaction, cognitive, language, and motor skills to a single future EF data point.

#### 2.2.4. Reliability

To establish the reliability of the coded data, 20% of the object interaction and Bayley-III videos were re-coded for intra- and inter-rater agreement. For the object interaction data, intra- and inter-agreements, calculated as [Agreed/(Agreed + Disagreed)] × 100, were 95.8 ± 5.5% and 93.0 ± 6.9%, respectively. For the Bayley-III, intra- and inter-rater agreements were calculated as ICC, resulting in ICC(3,1) = 100% and ICC(2,1) = 100%, respectively. For the MEFS, assessors completed a certification course to administer the MEFS App™ assessment. In the final module, assessors demonstrated their proficiency in administering a reliable and valid assessment by submitting a video for approval by the study’s lead examiners (RH, LYH).

### 2.3. Statistical Analyses

Statistical significance was evaluated at the level of *alpha* ≤ .05. First, Hierarchical Linear Modeling (HLM) [52] was performed to assess differences between the early developmental trajectories from baseline through 12 months post-baseline for the object interaction skills (*Unimanual Contacts*, *Bimanual Contacts*, *Looking*, *Looking During Unimanual Contacts*, and *Looking During Bimanual Contacts*) in children with low vs. high EF (*MEFS-C*) at 36 months post-baseline. Also, HLM was used to compare the early developmental trajectories from baseline through 12 months post-baseline for the Bayley-III scores (*Bayley-CG*, *Bayley-RL*, *Bayley-EL*, *Bayley-FM*, and *Bayley-GM*) in children with low vs. high EF (*MEFS-C*) at 36 months post-baseline. Although we had Bayley-III data at 36 months post-baseline, it was not included in these analyses, as the focus was on whether early (baseline to 12 months post-baseline) Bayley-III trajectories could predict later EF skills. HLM was used in both object interaction and Bayley-III outcome analyses to account for non-independence of observations in the current longitudinal data. Each object interaction skill and Bayley-III outcome was entered into the statistical models one at a time as a dependent variable. Linear and quadratic trends of change with age (*Age* and *Age^2^*, representing child’s prematurity-adjusted age in months) were entered into the models as Level-1 independent variables, whereas *MEFS-C* variable was entered into the models as a Level-2, grouping variable. This setup allowed for testing the significance of Age*MEFS-C and Age^2^*MEFS-C interactions. Random effects for intercept and *Age* slope were included in each model. Non-significant fixed and random effects were eliminated from the final models.

Additionally, Pearson correlation analyses (two-tailed, SPSS 29.0) were conducted to examine the relations between Bayley-III scores (*Bayley-CG*, *Bayley-RL*, *Bayley-EL*, *Bayley-FM*, and *Bayley-GM*) and children’s concurrent EF (*MEFS*) at 36 months post-baseline. Effect sizes for Pearson’s r were interpreted as small (~0.1), medium (~0.3), and large (>0.5) [53]. In contrast to above-mentioned HLM analyses that related early (baseline to 12 months post-baseline) developmental trajectories of object interaction and global development (Bayley-III) to EF using a dichotomous (low vs. high) classification of EF scores, these correlation analyses retained EF as a continuous variable, potentially providing new insights into the relations between concurrently measured developmental abilities and EF.

## 3. Results

### 3.1. Relations Between Object Interaction and Executive Function

**Figure 2:**
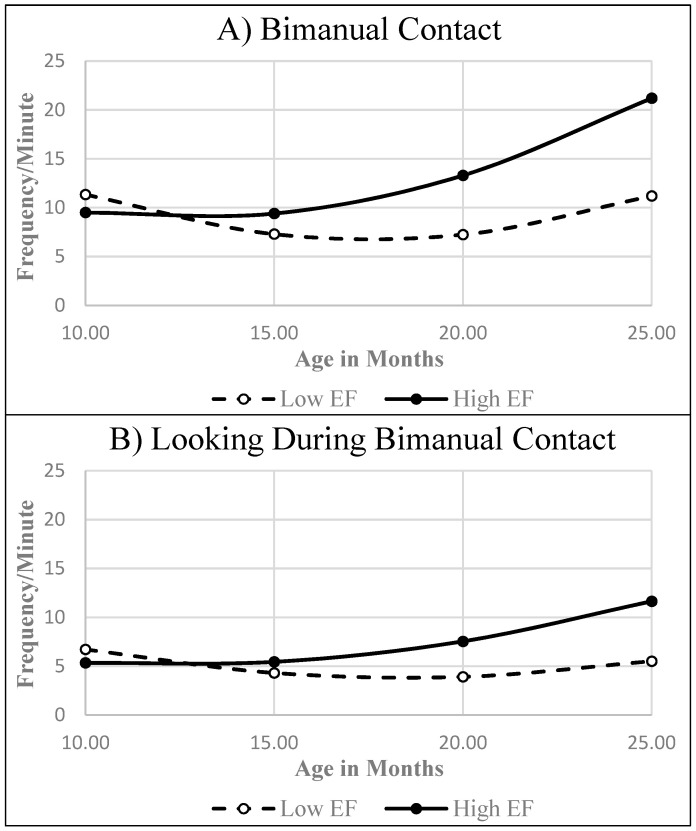
Estimated trajectories for the early object interaction skills found to differ between children with low vs. high EF at 36 months post-baseline.

Children with low vs. high MEFS scores at 36 months post-baseline differed significantly in their developmental trajectories for *Bimanual Contact* and *Looking During Bimanual Contact* but not for other object interaction variables (see Table 3). Modeled developmental trajectories suggested that from the age of about 12 months on, children later classified with high EF exhibited significantly more bimanual object contact and visual–bimanual activity than their peers with low EF (see Figure 2).

### 3.2. Relations Between Global Developmental Abilities and Executive Function

**Figure 3:**
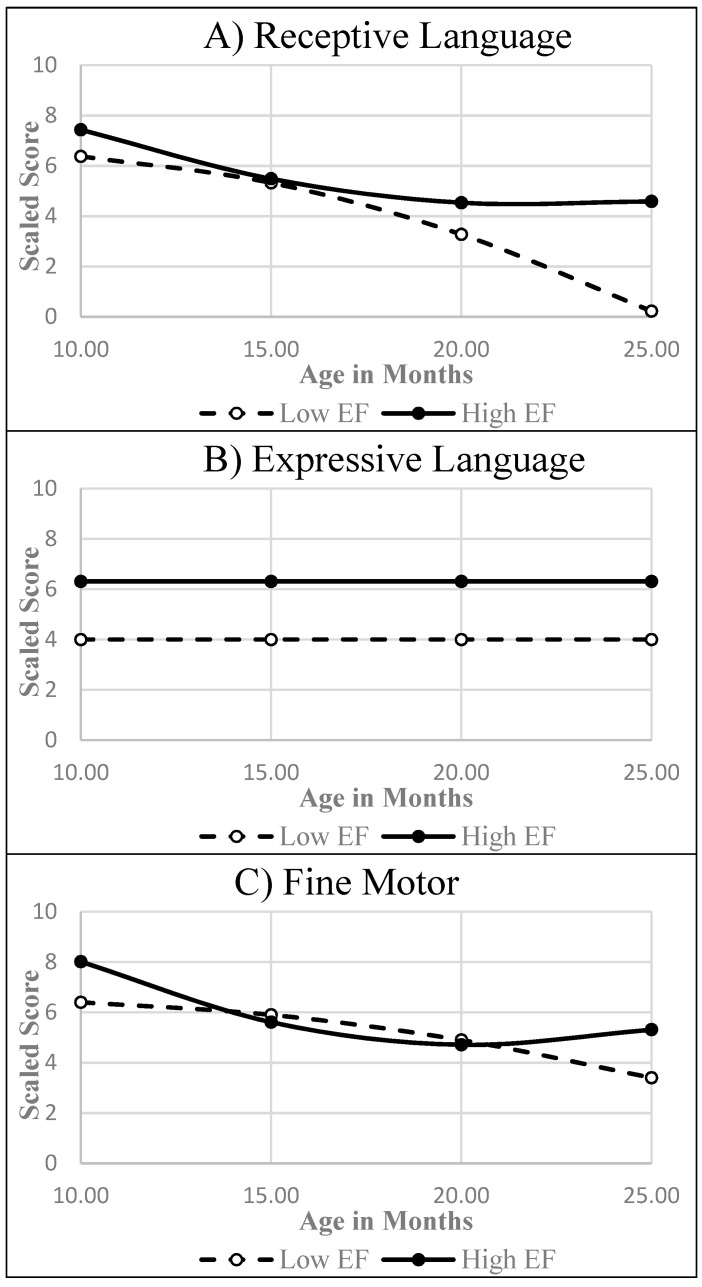
Estimated trajectories for the early Bayley-III areas of development found to differ between children with low vs. high executive function at 36 months post-baseline.

There were also significant differences in the early developmental trajectories for *Bayley-RL*, *Bayley-EL*, and *Bayley-FM* between children manifesting low vs. high EF at 36 months post-baseline (see Table 4). Modeled trajectories showed that, across the visits, children later identified as high EF scored higher than their peers with low EF in *Bayley-RL* and *Bayley-EL skills*; they also scored higher in *Bayley-FM* skills during the earlier (10–14 months) and later (20–25 months) periods of their development (see Figure 3). No differences were found between low vs. high EF children in their early trajectories of *Bayley-CG* or *Bayley-GM* skills.

Concurrent correlations between Bayley-III scores and MEFS scores at 36 months post-baseline showed significant positive correlations for *Bayley-CG scores* (*r*(11) = 0.68, *p* = .011, large effect), *Bayley-RL* scores (*r*(11) = 0.64, *p* = .019, large effect), *Bayley-EL* scores (*r*(11) = 0.71, *p* = .006, large effect), and *Bayley-FM* scores (*r*(11) = 0.70, *p* = .008, large effect), but not for *Gross Motor* scores (*r*(11) = 0.36, *p* = .251, medium effect).

## 4. Discussion

This study aimed to determine whether future EF skills in young children with motor delays are related to their early object interaction skills, as well as to their early and concurrent global development (i.e., cognitive, language, and motor skills).

### 4.1. Object Interaction and Executive Function

We hypothesized that early trajectories of object interaction skills (measured from baseline to 12 months post-baseline) would significantly relate to future EF (measured at 36 months post-baseline) in children with motor delays, as those early experiences provide the foundation for future problem-solving behaviors [27,29,54]. The current results showed that children with high EF exhibited more bimanual and visual–bimanual activity with objects than children with low EF. These findings align with previous research demonstrating that improvements in object interaction abilities (e.g., bimanual contact, open-handed contact, and palmar contact) and the amount of manual exploration (e.g., object rotating, fingering, and transferring) are positively related to future cognitive outcomes for preterm infants [27,55]. Additionally, preterm and full-term infants who showed greater exploration of objects in means–end tasks at 6 to 18 months of corrected age were more successful in problem-solving those tasks at the age of 2 years [32].

Importantly, bimanual and visual–manual object interaction requires complex temporal sequencing of performed actions and coordination between manual movements and visual perception; this is likely beneficial for training executive functions, such as attention, inhibition, shifting, and monitoring of performance [56,57,58]). Bimanual and visual–manual object interactions not only require high levels of coordination but also show goal-directedness. Previous research suggested that delays in goal-directed exploration of objects (e.g., purposeful actions aimed at solving a task) can hinder the development of key cognitive skills, including attention focus and anticipatory planning, while also affecting the ability to understand cause and effect in problem-solving tasks [32,33,59]. Therefore, we propose that bimanual and multimodal visual–manual behaviors may serve as key precursors for the development of EF skills, because they likely support the development of goal-directed behaviors that are fundamental for cognitive growth.

### 4.2. Global Developmental Abilities and Executive Function

We also hypothesized that children’s global developmental trajectories (i.e., cognitive, receptive language, expressive language, fine motor, and gross motor) during the first two years of life, as well as their development at around four years of age, would positively relate with their EF skills around four years of age. This hypothesis was made because early motor, language, and cognitive skills were found to be associated with EF skills at later ages [31,41,43,60,61].

This hypothesis was partially supported: children with high EF around four years of age had better early receptive language, expressive language, and fine motor abilities than children with low EF, but no significant relations were found for early cognitive or gross motor abilities. When comparing developmental abilities and EF at the same age (i.e., around four years), children’s EF positively related to their cognitive, expressive language, and fine motor abilities. These results align with previous research showing that children with better fine motor abilities at 1–2 years perform better on tasks related to EF (e.g., working memory and cognitive inhibitory control) at 3 years of age [30]. Also, a strong association between language and EF skills was found during the preschool and early school years, suggesting that children with language impairments may exhibit persistent deficits in EF tasks [43]. Thus, we conclude that early sensorimotor and communication behaviors may facilitate the development of key abilities foundational to the development of EF, including attention to people, objects, and events, goal-directedness, anticipatory motor control, planning of action sequences, understanding of the cause–effect concept, and problem-solving. These findings further support the theory of embodied cognition, which proposes that enhanced opportunities for sensorimotor experiences are fundamental to children’s cognitive development [24,54,59]

Interestingly, no relation was found between gross motor development and EF, despite previous evidence linking better motor skills to improved problem-solving in infancy and working memory in school-age children [37,38]. Gross motor skills, such as sitting, standing, walking, and balancing, involve broader, less precise movements and may not require the same level of fine-tuned executive control as fine motor tasks; therefore, their development may be less directly related to EF during early development. Additionally, since participants in the current study were enrolled based on developmental delays in the gross motor domain, the variability in this specific measure may have been limited in the sample, reducing the likelihood of detecting statistically significant relations.

No relation was found between early cognitive skills and future EF. It is important to note that early assessments of cognitive function heavily depend on children’s gross and fine motor skills. For example, a child’s performance on a means–end task (e.g., pulling a towel to obtain an out-of-reach toy supported on the towel) requires adequate postural control and manual function to initiate the reaching and pulling actions, even if the child understands the means–end relation between the towel and the toy [38]. The limited variability in motor skills within the current sample may have also resulted in reduced variability in the cognitive measures, potentially leading to the lack of significant findings.

### 4.3. Strengths, Limitations, and Future Directions

The strengths of the current study include multiple measures of child development, providing a comprehensive view of children’s motor and sensorimotor skills, language development, as well as cognitive and EF skills. Most of these skills were evaluated longitudinally, allowing for thorough assessment of developmental trajectories during early development.

Among the limitations, this study had a small sample size, which might limit the generalizability of the results to other children with motor delays. On the positive side, finding statistically significant relations in the context of this underpowered study not only provides valuable insights into the early development of EF but also underscores the need for further research in this area. 

The current study included only children with mild or moderate motor delays, as those with significant motor delays were unable to complete the EF tasks using the MEFS App™. Children with significant delays had deficits in motor skills necessary to engage in the game-based assessment of EF (e.g., trunk control, manual abilities, and visual–manual coordination), leading to their exclusion from the study. Future studies should evaluate larger samples of children with a broader range of abilities, possibly using other EF measures. 

### 4.4. Conclusions and Implications

Our exploratory results suggest that early bimanual and visual–bimanual interactions with objects, along with fine motor, cognitive, and language abilities, may serve as important precursors for the development of EF skills. This study provides valuable insights, such the potential for early detection of emerging EF deficits by identifying precursors like children’s manual and visual–manual abilities and global developmental skills. Recognizing these early indicators can support timely interventions aimed at proactive improvement of EF skills in at-risk populations. Clinicians may ensure more optimal development of EF in children with motor delays by targeting activities that focus on bimanual object interaction, visual–manual coordination, fine motor, cognitive, and language skills. Future research should investigate targeted interventions that focus on these abilities to enhance EF in young children with motor delays.

Evaluating early EF performance in young children and children with disabilities remains a challenge. Thus, a broader limitation in the field that must be addressed is the need for tools to evaluate EF in populations with cognitive or physical disabilities, as well as in typically developing children under the age of 2 years. Future research should focus on developing tools to support the evaluation of EF. It is crucial to understand the development of early EF skills not only in typically developing children but also in populations with delays and disabilities. Additionally, future studies should investigate these developmental relations across various contexts, including differing cultural and socioeconomic backgrounds.

## Figures and Tables

**Table 1:** Demographic and health-related information for children with low vs. high executive function, as well as the average across the entire sample (*Mean* ± *SD*).

Characteristic	Low EF	High EF	Total
Sample size	7	7	14
Male (%)	71.43	42.86	57.14
Gestational age at birth (weeks)	36.11 ± 6.26	38.76 ± 3.28	37.44 ± 5.00
Prematurity-corrected age at baseline (months)	11.57 ± 3.31	10.39 ± 1.34	10.98 ± 2.51
No specific diagnosis (%)	40.00	66.67	57.14
Diagnosis of cerebral palsy (%)	60.00	11.11	28.57
Diagnosis of congenital skeletal dysplasia or malformation (%)	0.00	22.22	14.29
Receiving early intervention services during the study (%)	41.67	58.33	85.71
African American race (%)	0	33.33	14.29
Caucasian race (%)	62.50	66.67	64.29
Other race (%)	37.50	0	21.42
Hispanic ethnicity (%)	62.50	0	35.71
Low SES (%) *	71.43	42.86	57.14
Middle-High SES (%) *	28.57	57.14	42.86
Mean MEFS at 36 months post-baseline	78.63 ± 14.72	100.83 ± 8.66	88.14 ± 16.60

*Note*. SES = socioeconomic status; EF = executive function; MEFS = Minnesota Executive Function Scale; % = percentage of participants falling into a specific category. * Participants’ SES was categorized based on the primary caregiver’s education (0 = less than high school; 1 = completed high school; 2 = more than high school) and poverty income ratio (PIR: the ratio of household income to the poverty level specific to the area of residence and household size, specified in the guidelines from the Department of Health and Human Services). Participants’ SES was defined as low when education was 0 and PIR < 2 and as middle-high when education ≥ 1 and PIR ≥ 2 [27].

**Table 2:** Assessment timeline (baseline and 3, 6, 12, and 36 months post-baseline); all time points at which a specific assessment was conducted are marked with an “X”.

Assessment	Assessment Timeline				
	Baseline	3 mos	6 mos	12 mos	36 mos
Object Interaction	X	X	X	X	
Bayley-III	X	X	X	X	X
Executive Function					X

**Table 3:** Detailed coding of behaviors in the object interaction assessment.

Object Interaction Assessment	
**Single Behaviors**	**Description**
Unimanual Contact	Instances when the child contacted the target object with only one hand
Bimanual Contact	Instances when the child contacted the target object with both hands
Looking	Instances when the child’s eyes were directed towards the target object
Multimodal behaviors *	
Looking During Unimanual Contact	Instances of the child looking at the object while contacting it with one hand
Looking During Bimanual Contact	Instances of the child looking at the object while contacting it with both hands

* Temporally overlapping occurrences of behaviors were identified using Filemaker Pro 19 software (Filemaker, Inc., Santa Clara, CA, USA) to form additional variables.

**Table 4:** Estimated statistical parameters for the fixed effects in the final multilevel HLM models relating the early trajectories for the developmental outcomes with the level of executive function (EF; low vs. high) at 36 months post-baseline; * statistically significant EF effects (*p* ≤ .05).

Outcomes	Statistical Parameters
***Object Interaction and Executive Function***	
Unimanual Contacts	*Intercept*: *β* = 19.50, *t*(13) = 11.78, *SE* = 1.66, *p* < .001 *EF*: *β* = −0.65, *t*(13) = −0.37, *SE* = 1.78, *p* = .720
Bimanual Contacts	*Intercept*: *β* = 31.44, *t*(13) = 4.76, *SE* = 6.60, *p* < .001 *EF*: *β* = −9.74, *t*(13) = −2.44, *SE* = 3.99, *p* = .030* Age: *β* = −2.81, *t*(54) = −3.53, *SE* = 0.80, *p* < .001 Age*EF: *β* = 0.79, *t*(54) = 4.00, *SE* = 0.20, *p* < .001* Age^2^: *β* = 0.08, *t*(54) = 3.50, *SE* = 0.02, *p* < .001
Looking	*Intercept*: *β* = 16.75, *t*(13) = 4.61, *SE* = 3.64, *p* < .001 *EF*: *β* = 1.05, *t*(13) = 1.29, *SE* = 0.82, *p* = .220 *Age*: *β* = −0.98, *t*(55) = −2.39, *SE* = 0.41, *p* = .020 *Age^2^*: *β* = 0.03, *t*(55) = 2.86, *SE* = 0.01, *p* = .006
Looking During Unimanual Contacts	*Intercept*: *β* = 12.52, *t*(13) = 9.69, *SE* = 1.29, *p* < .001 *EF*: *β* = −0.66, *t*(13) = −0.43, *SE* = 1.53, *p* = .673
Looking During Bimanual Contacts	*Intercept*: *β* = 17.51, *t*(13) = 3.16, *SE* = 5.55, *p* = .008 *EF*: *β* = −6.37, *t*(13) = −2.27, *SE* = 2.81, *p* = .041* Age: *β* = −1.48, *t*(54) = −2.26, *SE* = 0.65, *p* = .028 Age*EF: *β* = 0.50, *t*(54) = 3.60, *SE* = 0.14, *p* < .001* Age^2^: *β* = 0.04, *t*(54) = 2.25, *SE* = 0.02, *p* = .028
***Bayley-III and Executive Function***	
Cognitive Skills	*Intercept*: *β* = 13.17, *t*(11) = 5.31, *SE* = 2.48, *p* < .001 *EF*: *β* = 0.90, *t*(11) = 0.78, *SE* = 1.14, *p* = .450 *Age*: *β* = −0.95, *t*(37) = −3.90, *SE* = 0.24, *p* < .001 *Age^2^*: *β* = 0.02, *t*(37) = 3.60, *SE* = 0.01, *p* < .001
Receptive Language	*Intercept*: *β* = 5.48, *t*(11) = 2.84, *SE* = 1.93, *p* = .016 *EF*: *β* = 8.86, *t*(11) = 2.76, *SE* = 3.21, *p* = .019* Age: *β* = 0.29, *t*(35) = 1.05, *SE* = 0.28, *p* = .303 Age*EF: *β* = −1.18, *t*(35) = −2.60, *SE* = 0.45, *p* = .013* Age^2^: *β* = −0.02, *t*(35) = −1.80, *SE* = 0.01, *p* = .080 Age*EF^2^: *β* = 0.04, *t*(35) = 2.69, *SE* = 0.01, *p* = .011*
Expressive Language	*Intercept*: *β* = 4.00, *t*(11) = 5.87, *SE* = 0.68, *p* < .001 *EF*: *β* = 2.31, *t*(11) = 2.60, *SE* = 0.89, *p* = .025*
Fine Motor Skills	*Intercept*: *β* = 5.90, *t*(11) = 2.09, *SE* = 2.83, *p* = .061 *EF*: *β* = 11.41, *t*(11) = 2.04, *SE* = 5.60, *p* = .066 *Age*: *β* = 0.15, *t*(11) = 0.60, *SE* = 0.24, *p* = .563 Age*EF: *β* = −1.38, *t*(11) = −2.20, *SE* = 0.63, *p* = .050* Age^2^: *β* = −0.01, *t*(24) = −1.75, *SE* = 0.01, *p* = .093 Age*EF^2^: *β* = 0.04, *t*(24) = 2.35, *SE* = 0.02, *p* = .027*
Gross Motor Skills	*Intercept*: *β* = 2.36, *t*(11) = 1.87, *SE* = 1.26, *p* = .088 *EF*: *β* = 1.08, *t*(11) = 0.82, *SE* = 1.32, *p* = .429 *Age*: *β* = 0.08, *t*(38) = 2.28, *SE* = 0.04, *p* = .028

## Data Availability

The data that support the findings are available from the corresponding author upon request.
